# Short- and long-term effects of nutritional state on IGF-1 levels in nestlings of a wild passerine

**DOI:** 10.1007/s00442-023-05445-3

**Published:** 2023-09-07

**Authors:** Jaanis Lodjak, Jelle Boonekamp, Ádám Z. Lendvai, Simon Verhulst

**Affiliations:** 1 Department of Zoology, Institute of Ecology and Earth Sciences, University of Tartu, 2 Juhan Liivi Street, 50409 Tartu, Estonia; 2 Groningen Institute for Evolutionary Life Sciences, University of Groningen, Nijenborgh 7, 9747 AG Groningen, Netherlands; 3 Institute of Biodiversity, Animal Health & Comparative Medicine, College of Medical, Veterinary & Life Sciences, University of Glasgow, Glasgow, UK; 4 Department of Evolutionary Zoology and Human Biology, University of Debrecen, Debrecen, Hungary

**Keywords:** Adaptation, Fitness, Hormone, Somatic maintenance, Trade-off

## Abstract

Growth trajectories of young animals are intimately connected to their fitness prospects, but we have little knowledge of growth regulation mechanisms, particularly in the wild. Insulin-like growth factor 1 (IGF-1) is a central hormone in regulating resource allocation, with higher IGF-1 levels resulting in more growth. IGF-1 levels generally increase in conjunction with nutritional state, but whether IGF-1 levels are adjusted in response to current nutrient availability or to the nutrient availability integrated over a longer term is not well known. We tested for such effects by supplementary feeding the jackdaw (*Corvus monedula*) nestlings in experimentally reduced or enlarged broods with either water (control) or a food solution; these manipulations have long- and short-term effects on the nutritional state, respectively. Baseline plasma IGF-1 levels were higher in reduced broods. Food supplementation induced an increase in plasma IGF-1 levels measured one hour later, and this effect was significantly more substantial in nestlings in reduced broods. Changes in plasma IGF-1 levels increased with increased retention of the supplementary food, which was higher in reduced broods, explaining the stronger IGF-1 response. Thus, IGF-1 levels respond to short-term variations in the nutritional state, but this effect is amplified by longer-term variations in the nutritional state. We discuss our findings using a graphical model that integrates the results of the two treatments.

**Supplementary Information:**

The online version contains supplementary material available at 10.1007/s00442-023-05445-3.

## Introduction

Young vertebrates with determinate growth are in a race to achieve their optimal growth rate during the limited growing time in early life. How the growth rate is fine-tuned precisely is far from understood, but evidence indicates that insulin-like growth factor 1 (IGF-1) plays a central role. IGF-1 is a peptide synthesised in the liver in response to growth hormone (Dantzer and Swanson 2012; Lodjak and Verhulst 2020), and increased IGF-1 signalling is associated with accelerated growth and tissue differentiation (Le Roith et al. 2001; Dantzer and Swanson 2012) in every vertebrate class where this was studied (Dantzer and Swanson 2012; Lodjak and Verhulst 2020). When released to the blood stream, IGF-1 is bound to and carried around by IGF-binding proteins, which regulate the IGF-1 availability to its receptors (Hwa et al. 1999). Only a small fraction (up to 6%) of IGF-1 is in the free unbound fraction and has a biological effect upon binding to receptors (Allard and Duan 2018). Effects of IGF-1 on growth are manifested on the level of most, if not all, internal organs and external skin appendages (e.g. hair, feathers; Lupu et al. 2001; Trüeb 2018; Lodjak and Verhulst 2020; Lendvai et al. 2021). For example, an IGF-1 receptor^±^ knockout mutation reduced the asymptotic body mass of mice by two-thirds compared to the wild type (Baker et al. 1993), and daily injections of IGF-1 increased the growth of pied flycatcher (*Ficedula hypoleuca*) nestlings to a ~ 10% bigger body mass (Lodjak et al. 2017).

IGF-1 levels are highly nutrition dependent (Fontana et al. 2008; Lodjak and Verhulst 2020), with higher IGF-1 levels in nutritionally favourable setting, when both quantity and nutritional composition is considered, in species from humans and laboratory mice to fish, reptiles, birds, and mammals in the wild (Clemmons et al. 1985; Lodjak and Verhulst 2020). However, the temporal dynamics of the association between IGF-1 levels and nutritional state are still poorly understood, even in the best-studied species, humans and laboratory rodents. A recent meta-analysis showed that fasting induced a profound decrease in plasma IGF-1 levels within a day or several days in humans (Rahmani et al. 2019). However, energy restriction by itself tended to reduce plasma IGF-1 levels only when the restriction exceeded 50% of the daily energy requirement (Rahmani et al. 2019). Apparently, the suppressive effect of caloric restriction on circulating IGF-1 levels is less strong in humans compared to rodents (Fontana et al. 2008), possibly related to the higher ratio of energy reserves to energy turnover in larger species, but this hypothesis remains to be tested.

Free-living animals generally experience a stochastically fluctuating food supply, but we know very little about how this affects IGF-1 levels (Lodjak and Verhulst 2020; Tóth et al. 2022). The clearest examples of IGF-1 temporal long-term dynamics come from studies on wild-caught and aquaculture-raised teleost fish, where starvation for 3–4 weeks resulted in a significant drop in hepatic IGF-I mRNA levels and circulating IGF-1 levels (Duan and Plisetskaya 1993; Wilkinson et al. 2006). However, IGF-I mRNA levels in individual tissues (e.g. spleen, kidneys, gut) did not noticeably decrease (Duan and Plisetskaya 1993). Interestingly, a week-long refeeding of starved teleost fish did not change IGF-1 levels back to baseline values (Wilkinson et al. 2006). Thus, circulating IGF-1 levels appear more susceptible to impairment in the nutritional state than tissue levels. Effects of nutrition on plasma IGF-1 have also been shown in the wild great tit (*Parus major*) nestlings using brood size manipulation, which typically affects the nutritional state. When nestlings grew in experimentally reduced broods, which receive more per capita food than nestlings in enlarged broods, they had higher IGF-1 levels (Lodjak et al. 2014). However, whether this reflects a difference in the likelihood that they were fed shortly before sampling, i.e. a short-term effect or a more permanent (long-term) effect of the manipulation, is an open question that we address in the present study.

To study the temporal link between food and circulating IGF-1, we experimentally manipulated the long-term nutritional state of altricial nestlings via brood size manipulation and short-term nutritional state through food supplementation to test two predictions. Firstly, we predicted that circulating IGF-1 levels increase following food supplementation. Secondly, we predicted that circulating baseline IGF-1 levels would be higher in nestlings in reduced broods, given that brood size manipulation has a permanent effect on the provisioning rate of nestlings, as observed directly (Lessells 1993) and reflected in growth and IGF-1 levels in earlier studies (Lodjak and Verhulst 2020). Lastly, we tested for an interaction between food supplementation and brood size manipulation, for which we have no directional prediction, as the interaction, when it exists, could go either way. For example, the circulating IGF-1 response to supplementary food could be stronger in enlarged broods when nestlings in reduced broods are already growing at their optimal rate, or the circulating IGF-1 response could be weaker in enlarged broods when these nestlings allocate a more significant proportion of the supplementary food to restoring energy reserves, leaving fewer resources for growth.

## Methods

### Study system

We studied free-living jackdaws (*Corvus monedula*), a semi-colonial cavity-nesting corvid species, near Groningen, the Netherlands (53.17°N, 6.61°E) from April to June of 2018. Nest boxes were grouped into six colonies with 120 boxes in total with an average occupancy rate exceeding 90%. All colonies were situated in a structurally very similar landscape. We established the first egg's laying date, clutch size, and hatch date through regular nest checks. On day 5 of the oldest nestling in the brood (hatching = day 1), a brood size manipulation was conducted as previously described as part of a longitudinal study (see details in Boonekamp et al. 2014). Briefly, using two age-matched broods, we randomly selected three nestlings from the brood to be reduced, and moved these to the brood to be enlarged, and we returned one nestling from this brood to the reduced brood. Therefore, the net manipulation was −2 or +2 nestlings. Note that to increase statistical power, there was no control group.

Mean clutch size, brood size at hatching, and post-manipulation brood sizes at days 5 and 15 were for the treatments as follows: reduced (food group: 4.82 (SD = 0.40), 4.18 (SD = 0.98), 2.27 (SD = 0.79), 2.09 (SD = 0.70); control group: 4.57 (SD = 1.27), 3.29 (SD = 0.95), 1.71 (SD = 0.49), 1.57 (SD = 0.53)); enlarged broods (food group: 4.19 (SD = 0.68), 2.48 (SD = 1.01), 4.63 (SD = 0.84), 4.63 (SD = 0.84); control group: 4.73 (SD = 0.46), 3.91 (SD = 1.27), 5.77 (SD = 1.31), 4.91 (SD = 1.63)). At age 15 days, the difference in brood size is less than the initial manipulation effect due to higher mortality in enlarged broods.

### Food supplementation

The food supplementation experiment was conducted on nestling day 15 (jackdaw nestlings fledge when > 30 days old; see Fig. S1 in Supplementary Information) in a subset of the study population. We randomly assigned both broods of an age-matched dyad of a reduced and an enlarged brood to either the fed or the control group—all nestlings in a brood received the same treatment. Body mass of nestlings prior to the experiment did not differ between the treatment groups (reduced broods: *t* = 0.79, *p* = 0.43 (food group: 191 g (SE = 7.6 g), control group: 199 g (SE = 9.1 g)); enlarged broods: *t* = 1.67, *p* = 0.10 (food group: 182 g (SE = 5.8 g), control group: 193 g (SE = 6.3 g))). Six dyads (11 nestlings in reduced broods, 27 nestlings in enlarged broods) received food supplementation, and six dyads (7 nestlings in reduced broods, 22 nestlings in enlarged broods) received the control treatment. In the supplemented group, we fed each nestling with 3 g of NutriBird A21 (Versele-Laga, Belgium; see Supplementary Information for nutritional composition) hand-rearing food powder dissolved in 9 g of water. Control nestlings received only 9 g of water. Food supplementation was done individually with a syringe with a soft plastic tube attached. Feeding was conducted each day at a random time between 10:00 and 14:00 h.

Blood sampling for IGF-1 measurements took place immediately prior to feeding and 60 min after feeding. The period of 60 min was chosen based on the published digestive retention time estimates for the feeding solutions (McWhorter et al. 2009). During the 1 h period between baseline and follow-up sample, chicks were in their nest, allowing the parents to continue feeding. Samples (up to ~ 120 µl) were taken from the brachial vein and stored at + 4^○^C until centrifuged at 8000G for 8 min within hours to separate plasma from cells. Plasma was stored at −20 ^○^C until analysis for maximum of 6 months. Nestlings were weighed with a digital scale to the nearest 1.0 g to obtain pre-treatment (before feeding), post-feeding (directly after feeding), and post-treatment (1 h after feeding) body mass values.

### Hormonal analysis

IGF-1 that is not bound to IGF-1 binding proteins is called a free fraction; this part of the hormone is also the biologically active fraction and is an established nutritional marker in clinical studies (Janssen et al. 2003). In the current study, we were interested in the biologically active fraction of the hormone and therefore did not carry out the extraction protocol on the blood plasma to avoid adding the bound fraction from the carrier proteins into our analysis.

IGF-1 levels were measured in duplicate in 20 μl of plasma using an in-house ELISA described previously (Mahr et al. 2020). We used jackdaw and pre-validated chicken plasma to determine intra- (jackdaw: 4.8%) and inter-assay coefficient of variations (jackdaw: 9.7%). Cross-reactivity with IGF-2 and insulin was less than 1%. We validated the assay for the jackdaw by showing the parallelism between serially diluted samples and the standard curve. All samples were analysed within a few days of each other; therefore, storage time at −20 ^○^C did not differ between the samples, and all samples were thawed only once.

### Statistical analysis

Using two general linear mixed models, we investigated how body mass and IGF-1 levels changed (factor ‘phase’ with two levels: pre-treatment and post-treatment) in response to food provisioning (factor ‘provisioning’ with two levels: treatment and control) and whether these changes differed between nestlings in reduced and enlarged broods (factor ‘brood size’ with two levels). Levels of all three fixed factors were coded as 0 and 1 and mean-centred prior to analysis to allow meaningful interpretation of main effects while being part of interactions also included in the model. With respect to these fixed factors, we fitted a full model, i.e. with three-way interaction and all lower-order interactions. NestlingID and dyadID (see above) were included as random intercepts, with nestlingID nested in dyadID. Hormonal assayID explained an insignificant amount of information throughout the analyses and was therefore omitted from all models.

Additionally, we calculated differences in IGF-1 levels and body mass by subtracting pre-treatment values from post-treatment values (i.e. one hour after supplementary feeding). We ran a general linear mixed model to analyse how the change in body mass was associated with the corresponding change in IGF-1 levels in brood size manipulation groups. DyadID was also included as a random intercept in this model. Levels of each of the two fixed factors were also coded as 0 and 1 and mean-centred prior to analysis. Sex (based on molecular analysis) was included in the models, since jackdaw nestling body mass is sex dependent, with males being larger. Data were analysed using R v3.6.1 (R Development Core Team 2020) statistical software, using packages *lmerTest* (Kuznetsova et al. 2015) and lme4 (Bates et al. 2014) for models, and *emmeans* (Lenth et al. 2018) for Tukey HSD post-hoc analysis. Satterthwaite's method was used to approximate the degrees of freedom in all models. IGF-1 was log_e_-transformed before analyses to meet the model assumptions.

## Results

**Fig. 1:**
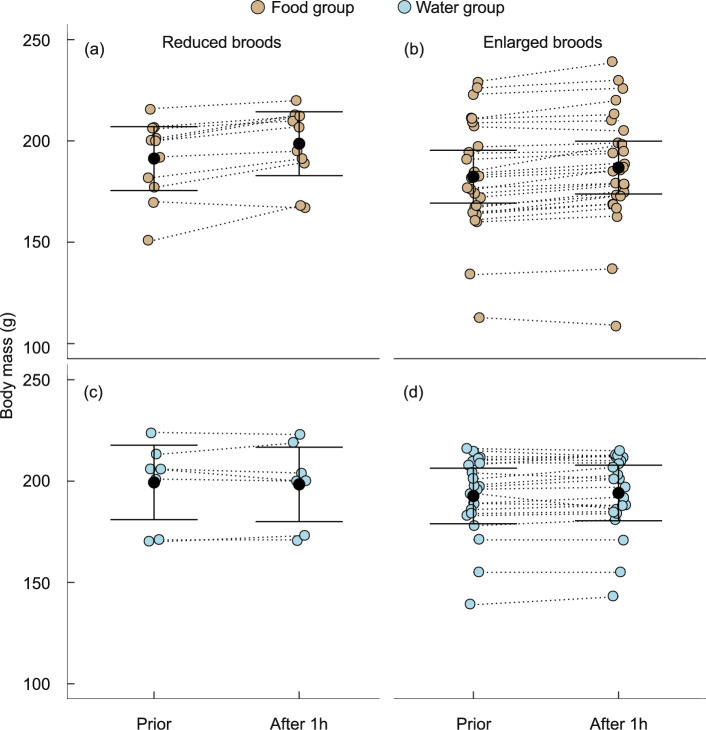
Body mass before and 1 h after supplementation of nestlings with food (**a**, **b**) or water (control) groups (**c**, **d**) in reduced and enlarged broods. Whiskers denote 0.95 confidence limits of the marginal mean estimate. Dotted lines connect data of individual nestlings

Body mass was 8 g lower in enlarged broods as expected (Boonekamp et al. 2014), but this difference did not reach statistical significance in the current (modest) data set (Table 1a; post-hoc: *t* = 1.28, df = 61.50, *p* = 0.20). Body mass did not differ between the provisioning groups prior to the treatments (post-hoc: *t* = 1.53, df = 60.20, *p* = 0.13). Compared to pre-feeding mass, body mass was significantly increased 1 h after receiving food compared to nestlings being given water only (Fig. 1), presumably because digesting food takes longer than digesting water. However, the magnitude of the body mass increase differed significantly between reduced and enlarged broods (*t* = 2.18, df = 32.38, *p* = 0.04). Nestlings in the reduced broods gained ~ 63% more mass (7.32 ± 1.17 g (SE); Fig. 1) over the 1 h period after receiving supplementary food compared to food-supplemented nestlings in enlarged broods (4.50 ± 0.75 g; Fig. 1). Thus, as intended, both experimental treatments (brood size manipulation and supplementary feeding) induced a change in nutritional state, setting the stage to investigate effects on IGF-1.

**Table 1:** Summary statistics of the model of log IGF-1 levels and body mass in response to food provisioning (water (control) = 0; food = 1), manipulated brood size (reduced = 0, enlarged = 1) and experimental phase (pre-treatment = 0, post-treatment = 1)

(a) Body mass (untransformed)					
Fixed effects	Estimate	SE	df	*t*	*p*
Intercept	190.63	4.90	4.93	38.91	< 0.001
Phase	3.34	0.48	62.00	7.02	< 0.001
Supplementation	−7.49	5.25	59.74	1.43	0.16
Brood size	−8.25	5.91	60.38	1.40	0.17
Sex	1.44	0.75	63.22	1.93	0.06
Phase × Supplementation	4.44	0.96	62.00	4.62	< 0.001
Brood size × Phase	−0.54	1.13	62.12	0.48	0.63
Brood size × Supplementation	−4.92	11.83	59.00	0.42	0.68
Brood size × Phase × Supplementation	−5.27	2.26	62.08	2.33	0.02

Random effects	Variance
nestlingID: dyadID	430.58
dyadID	103.90
Residual	7.50
*R*^2^_conditional_	0.99
*R*^2^_marginal_	0.06

(b) IGF-1 (log-transformed)					
Fixed effects	Estimate	SE	df	*t*	*p*
Intercept	2.92	0.04	5.75	71.44	< 0.001
Phase	0.21	0.04	67.21	5.00	< 0.001
Supplementation	0.03	0.05	63.49	0.66	0.51
Brood size	−0.14	0.06	65.15	2.48	0.02
Sex	0.03	0.05	128.90	0.56	0.57
Phase × Supplementation	0.37	0.09	67.24	4.31	< 0.001
Brood size × Phase	−0.04	0.10	70.60	0.40	0.69
Brood size × Supplementation	−0.34	0.11	63.45	2.97	0.004
Brood size × Phase × Supplementation	−0.28	0.20	69.51	1.42	0.16

Random effects	Variance
nestlingID: dyadID	0.01
dyadID	0.006
Residual	0.06
*R*^2^_conditional_	0.45
*R*^2^_marginal_	0.30

All predictors were mean-centred for ease of interpretation of main effects and interaction terms

**Fig. 2:**
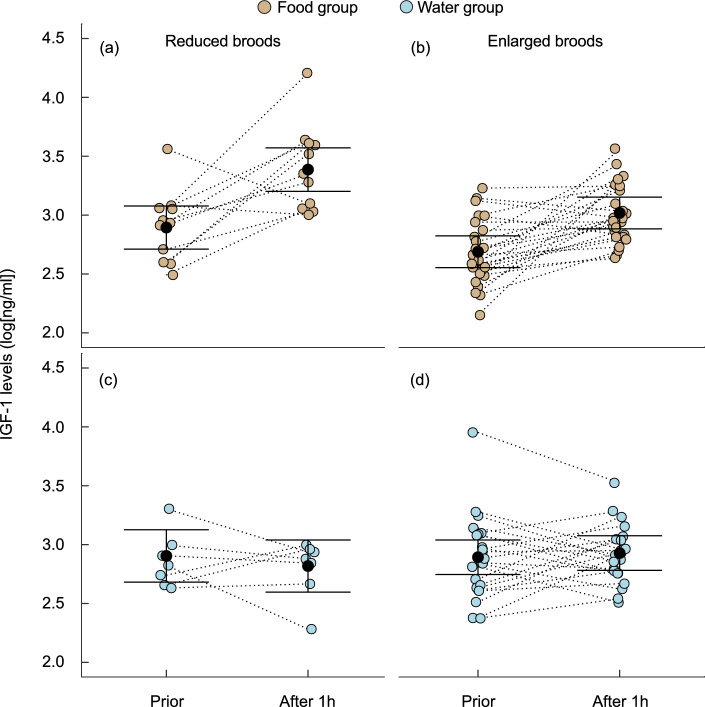
Levels of IGF-1 before and 1 h after supplementation of nestlings with food (**a**, **b**) or water (control) groups (**c**, **d**) in reduced and enlarged broods. Whiskers denote 0.95 confidence limits of the mean. Dotted lines connect data of individual nestlings

IGF-1 levels were significantly elevated 1 h after receiving food (Fig. 2), as evidenced by the significant supplementation by phase interaction (Table 1b). Nestlings in reduced broods had higher IGF-1 levels (Table 1b). There was a significant interaction between brood size manipulation and food supplementation, indicating a stronger effect of provisioning in reduced broods (Table 1b). On the other hand, the three-way interaction between phase, brood size manipulation, and food supplementation did not reach significance. This contrasts with the significant interaction between brood size and supplementation (*t* = 2.97, df = 63.45, *p* = 0.004; Table 1b), because there was no difference between supplemented and control nestlings prior to supplementation (*t* = 1.37, df = 118.00, *p* = 0.17). To resolve this we performed a post-hoc test on the supplemented broods only which revealed that the effect of food supplementation on IGF-1 differed between nestlings in the reduced and enlarged broods (*t* = 2.98, df = 28.71, *p* = 0.006), with nestlings in the reduced broods showing a larger increase (35%; 15.12 ± 3.25 ng/ml; Fig. 2) compared to nestlings in enlarged broods (21%; 3.92 ± 2.46 ng/ml; Fig. 2).

**Fig. 3:**
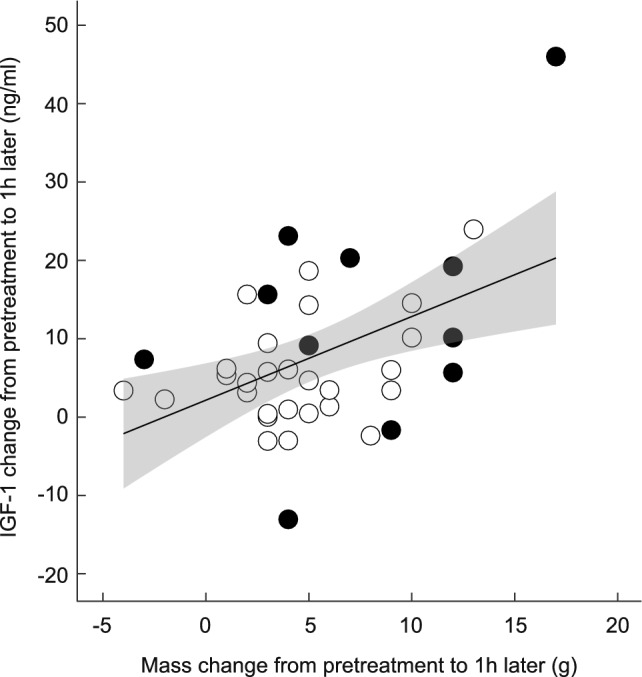
Association between changes in IGF-1 levels and body mass. Changes in both traits were computed between pre-feeding values and respective values 1 h later. The sample size is 38 food-supplemented nestlings. The grey area denotes 0.95 confidence limits for the regression fit. Filled circles denote nestlings in the enlarged broods, and empty circles denote nestlings in the reduced broods

Given that the effects of supplementary feeding on both mass and IGF-1 were stronger in reduced broods, we hypothesised that these responses might be mechanistically linked, mediating the interactions with brood size. Indeed, among nestlings that received supplementary food, the mass change over the 1 h period was positively associated with the IGF-1 level change within that time period (*t* = 3.19, df = 36.00, *p* = 0.003, *R*^2^ = 0.22; Fig. 3), and brood size manipulation (*p* = 0.25) and its interaction with mass change (*p* = 0.45) did not explain a significant amount of the residual variation.

## Discussion

We compared responses to a short-term and a longer-term manipulation of the nutritional state on IGF-1 levels in growing nestlings to investigate the association between nutritional state and IGF-1 levels and the temporal dynamics of this association. The short-term manipulation (food provisioning) of the nutritional state strongly increased IGF-1 levels. In general terms, this finding is in line with the widely documented association between IGF-1 and nutritional state (Schew et al. 1996; Duncan et al. 2015; Tóth et al. 2022). Our findings extend this insight by showing that this process is highly dynamic, with a strong IGF-1 response to short-term changes in resource availability. This characteristic of IGF-1, alongside its short half-life (in the order of hours), makes IGF-1 an excellent tool for nestlings to regulate their resource allocation to growth in response to fluctuations in the nutritional state (Clemmons et al. 1985; Caregaro et al. 2001; Ley et al. 2013). We used a feeding solution rich in both calories and nutrients, and either of these components could have independent positive effects on IGF-1 levels (e.g. Fontana et al. 2008). Further experimentation is required to separate the effects of caloric intake from the effects of specific nutrients on IGF-1.

Manipulation of brood or litter size generally affects the nutritional state of offspring, as evidenced by the effect this manipulation generally has on growth (e.g. Sanz and Tinbergen 1999; Pettifor et al. 2001; Neuenschwander et al. 2003), also in our population of jackdaws (Boonekamp et al. 2014). In agreement with the general dependence of IGF-1 levels on nutritional state, an earlier investigation reported a negative association between manipulated brood size and nestling IGF-1 (Lodjak et al. 2014). We here provide the first replication of this result and note that this effect can have arisen in different ways. On average, at the time of sampling, nestlings in reduced broods are more likely to have been recently fed than nestlings in enlarged broods, and the observed brood size effect on IGF-1 levels may reflect this short-term effect only. Alternatively, but not mutually exclusive, nestlings in reduced broods may have higher IGF-1 levels permanently, because independent of the level of recent feeding, they are in a better nutritional state. Our experiments indicate that both processes contribute to the brood size effect on IGF-1 levels, since the supplementary feeding induced a larger IGF-1 increase in reduced broods compared to enlarged broods.

**Fig. 4:**
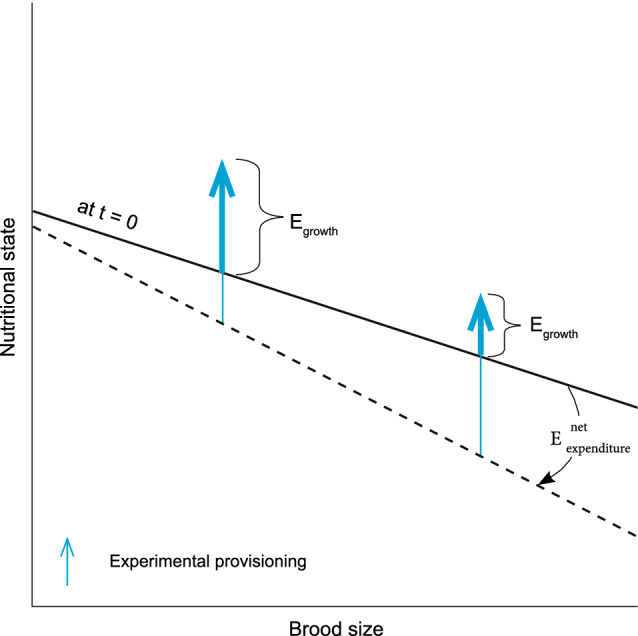
A graphic model integrating our experimental findings. The solid line represents the nutritional state (energy level) at the start of the supplementary feeding (*t* = 0). The dashed line represents the shifted nutritional state due to energy lost during the ‘experimental hour’. Vertical arrows denote the energy gain via food supplementation, with the bold part of the vertical arrows denoting the energy retained and hence available for somatic growth after ‘the experimental hour’, while the thin part of the arrow represents the supplemental mass that was lost over the ‘experimental hour’. Mass retained was proportional to the change in IGF-1 levels (Fig. 3)

We hypothesise that the stronger IGF-1 response to supplementary feeding in reduced broods was due to these nestlings being in a better nutritional state when they received the supplementary food, which allowed them to retain a larger proportion of the supplementary food to growth (Fig. 4). This interpretation was inspired by the mass changes we recorded, which showed that nestlings in reduced broods retained more of the supplementary mass gained by feeding compared to nestlings in enlarged broods. The body mass change is the net outcome of several factors, including parental provisioning, digestive efficiency, and energy spent on somatic maintenance during the hour between the initial and final sample, which in this study, we cannot distinguish. However, given that actual growth over the one-hour period has to be negligible, the difference in mass change implies that nestlings in enlarged broods had fewer resources remaining for growth, in agreement with the diminished IGF-1 response. This interpretation is supported by the analysis of variation within the group of nestlings receiving supplementary food, which revealed that mass change over the one-hour period between baseline and follow-up sampling explained variation in IGF-1 response to supplementary feeding. Indeed, when the mass change was controlled for statistically, the brood size manipulation did not explain a significant part of the variation, although there was still a trend for such an effect. This finding agrees with the graphic model we developed to summarise our study (Fig. 4).

In conclusion, IGF-1 has been portrayed as a key hormone mediating resource allocation at multiple life stages (Swanson and Dantzer 2014; Lewin et al. 2017; Lodjak et al. 2018; Montoya et al. 2022), and our results are in line with this view. Resource allocation needs to be regulated on different time scales due to factors affecting parental provisioning on different time scales. For example, the quality of the habitat, parental foraging proficiency, and the number of siblings will affect the provisioning rate permanently, while climatic conditions and other factors with immediate effects on prey availability will cause short-term (unpredictable) variations in the provisioning rate. It appears that IGF-1 performs both functions, responding to short- and long-term provisioning-induced variation in the nutritional state, likely mediating associated variation in growth. Our focus was on free IGF-1 level temporal dynamics, but the overall picture can be broadened by considering carrier proteins and receptors on the one hand, and IGF-1- and food-induced behavioural variation on the other hand. This conceptual framework is still poorly understood and exploring could stand as a roadmap for future studies.

### Supplementary Information

Below is the link to the electronic supplementary material.

Supplementary file1 (PDF 171 KB)

## Data Availability

The data analysed in this study is made available by the corresponding author upon reasonable request.
