# Supplementary material for: Short- and long-term effects of nutritional state on IGF-1 levels in nestlings of a wild passerine
Source: Oecologia. 2023 Sep 7;203(1-2):27–35. doi: 10.1007/s00442-023-05445-3 (PMC10615909; doi:10.1007/s00442-023-05445-3)
Supplement: Supplementary file 1 — Supplementary file1 (PDF 171 KB) [file 442_2023_5445_MOESM1_ESM.pdf]

Supplementary material

Short- and long-term effects of nutritional state on IGF-1 levels in nestlings of a wild passerine

Jaanis Lodjak[1,2,*], Jelle Boonekamp[2,4], Ádám Z. Lendvai[3], Simon Verhulst[2]

[1] Department of Zoology, Institute of Ecology and Earth Sciences, University of Tartu, 2 Juhan Liivi Street, Tartu 50409, Estonia.

[2] Groningen Institute for Evolutionary Life Sciences, University of Groningen, Nijenborgh 7, 9747 AG Groningen, Netherlands.

[3] Department of Evolutionary Zoology and Human Biology, University of Debrecen, Debrecen, Hungary.

[4] Institute of Biodiversity, Animal Health & Comparative Medicine, College of Medical, Veterinary & Life Sciences, University of Glasgow, Glasgow, UK

VERSELE-LAGA Nutribird A21 nutritional composition (data retrieved from the producer)

COMPOSITION

bakery products, vegetable protein extracts, oils and fats, derivatives of vegetable origin, minerals, sugars, yeasts, organic acids (2500 mg/kg), psyllium, mannan-oligosaccharides (1000 mg/kg), lecithin

ANALYTICAL CONSTITUENTS

protein 21%, fat content 9%, crude ash 9.0%, crude fibre 1.5%, lysine 1.25%, methionine 0.6%, tryptophan 0.27%, threonine 0.8%, calcium 1.15%, phosphorus 0.8%, sodium 0.4%, magnesium 0.14%

ADDITIVES/KG

NUTRITIONAL ADDITIVES

vitamin A 7200 IU, 3a160a β-carotene 4.35 mg, vitamin D3 1500 IU, vitamin E 85 mg, vitamin B1 7.2 mg, vitamin B2 16 mg, calcium-D-pantothenate 20 mg, vitamin B6 6 mg, vitamin B12 0.03 mg, vitamin C 52 mg, niacin 80 mg, folic acid 1.5 mg, biotin 0.26 mg, choline chloride 700 mg, 3b202 (iodine) 2 mg, 3b405 (copper) 10 mg, 3b503 (manganese) 100 mg, 3b605 (zinc) 96 mg, 3b802 (selenium) 0.1 mg, 3b811 (organic selenium) 0.1 mg

ZOO TECHNICAL ADDITIVES

bacillus subtilis C-3102 (DSM 15544) 22.10 8 CFU, endo-1,4-β-xylanase (EC 3.2.1.8) 3000 EPU

TECHNOLOGICAL ADDITIVES

antioxidants

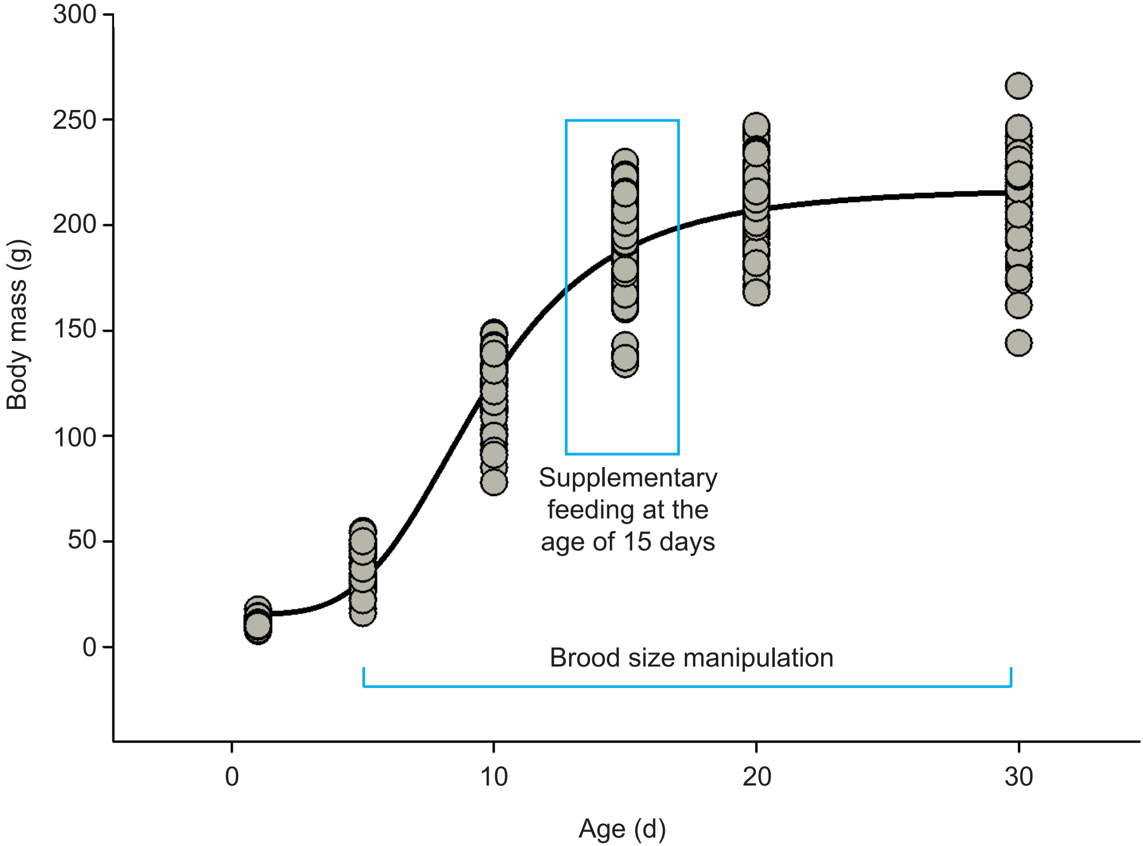

Figure S1. Growth curve of studied jackdaw (*Corvus monedula*) nestlings. The rectangle highlights the age when the supplementary feeding experiment was conducted. The line above the x-axis indicates the duration of the brood size manipulation between the ages of 5 and 30 days. The solid black line denotes the 4-parameter logistic curve, and gray dots are the raw measures of body mass.
